# Endothelial progenitor cells in acute ischemic stroke

**DOI:** 10.1002/brb3.175

**Published:** 2013-09-22

**Authors:** Joan Martí-Fàbregas, Javier Crespo, Raquel Delgado-Mederos, Sergi Martínez-Ramírez, Esther Peña, Rebeca Marín, Lavinia Dinia, Elena Jiménez-Xarrié, Ana Fernández-Arcos, Jesús Pérez-Pérez, Luis Querol, Marc Suárez-Calvet, Lina Badimon

**Affiliations:** 1 Department of Neurology, IIB Biomedical Research Institute, Hospital de la Santa Creu i Sant Pau08025, Barcelona, Spain; 2 Cardiovascular Research Center, IIB-Sant PauAvda Sant Antoni M.Claret, 167, 08025, Barcelona, Spain

**Keywords:** Endothelial progenitor cells, ischemic stroke, statin

## Abstract

**Objectives:**

The levels of circulating endothelial progenitor cells (EPCs) in ischemic stroke have not been studied extensively and reported results are inconsistent. We aimed to investigate the time course, the prognostic relevance, and the variables associated with EPC counts in patients with ischemic stroke at different time points.

**Material and methods:**

We studied prospectively 146 consecutive patients with ischemic stroke within the first 48 h from the onset of symptoms (baseline). We evaluated demographic data, classical vascular risk factors, treatment with thrombolysis and statins, stroke etiology, National Institute of Health and Stroke Scale score and outcome (favorable when Rankin scale score 0–2). Blood samples were collected at baseline, at day 7 after stroke (*n* = 121) and at 3 months (*n* = 92). The EPC were measured by flow cytometry.

**Results:**

We included 146 patients with a mean age of 70.8 ± 12.2 years. The circulating EPC levels were higher on day 7 than at baseline or at 3 months (*P* = 0.045). Pretreatment with statins (odds ratio [OR] 3.11, *P* = 0.008) and stroke etiology (*P* = 0.032) were predictive of EPC counts in the baseline sample. EPC counts were not associated with stroke severity or functional outcome in all the patients. However, using multivariate analyses, a better functional outcome was found in patients with higher EPC counts in large-artery atherosclerosis and small-vessel disease etiologic subtypes.

**Conclusions:**

After acute ischemic stroke, circulating EPC counts peaked at day 7. Pretreatment with statins increased the levels of EPC. In patients with large-artery atherosclerosis and small-vessel disease subtypes, higher counts were related to better outcome at 3 months.

## Introduction

Asahara et al. (1997) described endothelial progenitor cells (EPC) in human peripheral blood. EPC are immature endothelial circulating cells mobilized from the bone marrow. These cells are involved in repairing the damaged endothelium and in facilitating neovascularization after ischemia (Asahara et al. 1997; Urbich and Dimmeler 2004; Fadini et al. 2007; Rouhl et al. 2008).

The role of EPC in health and disease is not understood completely. Most studies of healthy subjects and patients with coronary artery disease (CAD) report that the number and function of circulating EPC decrease with age and with the presence of classical vascular risk factors (Hristov and Weber 2004; Fadini et al. 2007). Also, EPC levels (counts) increase after an ischemic event and a low number of EPC predict a higher frequency of vascular events during follow-up in healthy subjects (Hill et al. 2003) and in patients with CAD (Werner et al. 2005). These studies suggest that EPC play an important role in the risk of vascular events and in vascular homeostasis.

EPC counts have not been studied frequently in patients with ischemic stroke, and the results are conflicting. Some studies (Ghani et al. 2005; Chu et al. 2008; Zhou et al. 2009) reported lower counts of EPC in patients in the acute stage of ischemia compared to controls, while other studies (Dunac et al. 2007; Yip et al. 2008, 2011; Navarro-Sobrino et al. 2010) reported the opposite. Moreover, higher EPC levels have been associated with a favorable short and long-term outcome in some studies (Sobrino et al. 2007; Yip et al. 2008; Taguchi et al. 2009). Unfortunately, these investigations did not focus on the variables associated with the EPC counts and did not evaluate the significance of stroke etiology. It seems that evaluation of these aspects is necessary to ascertain the therapeutic and prognostic relevance of this population of cells in patients with cerebral ischemia. Therefore, our study investigated systematically the EPC counts in the acute, subacute, and chronic stages of ischemic stroke of different etiologies, the associated variables, and their prognostic value.

## Materials and Methods

### Patients

We prospectively studied consecutive patients with a suspected ischemic stroke that were admitted to the Neurology Department at our Hospital. All the patients were included within the first 48 h after the onset of stroke. The Ethics Committee at Hospital de la Santa Creu i Sant Pau (Barcelona, Spain) approved the study, and written informed consent was obtained from participating patients or their legal representatives. Exclusion criteria were as follows: a previous modified Rankin scale score higher than 2; a National Institute of Health and Stroke Scale (NIHSS) score of 0; the lack of processing of the blood sample within 30 min after extraction, as this was the predefined time window to obtain reliable results. Because our laboratory could process the blood samples only during working days, we excluded those patients admitted during the weekend in whom the sample could not be obtained before the 48-h limit.

### Endothelial progenitor cells measurement

Blood samples (4 mL) were obtained by venopuncture and collected in ethylene diamine tetra acetic acid (EDTA) tubes at three time points: baseline (within 48 h from the onset of stroke), and 7 and 90 days after the onset of stroke. Identification of EPC is typically based on the cell surface expression of the protein. It is well established that EPC are positive for the following three surface antigens: CD34 (a marker of hematopoietic stem cells), CD133 (a marker of immature hematopoietic stem cells), and KDR (a marker of endothelial protein) (Urbich and Dimmeler 2004; Werner and Nickenig 2006; Lembo et al. 2012; Paczkowska et al. 2013). We analyzed EPC by flow cytometry as previously described (Rustemeyer et al. 2006). In brief, in order to lyse erythrocytes the EDTA-blood samples were treated with BD Pharm Lyse™ lysing solution (BD Biosciencie, San Jose). Then nucleated cells were stained with a phycoerythrincyanin-conjugated anti-CD34 monoclonal antibody (Beckman-Coulter, Marseille, France), phycoerythrin-conjugated anti-CD133 monoclonal antibody (Miltenyi-Biotec, Bergisch-Gladbach, Germany), and carboxyfluorescein-conjugated anti-KDR monoclonal antibody (R&D Systems, Wiesbaden, Germany). Isotype-matched antibodies were used as controls. After staining, the samples were fixed with 0.2% formaldehyde for 2 h and then analyzed by flow cytometry (EPICS XL). We settled on the appropriate gate for mononuclear cells based scattering light properties. Typically 300,000 total events were acquired to determinate the percentage of the CD34+/VEGF-R2+/CD133+ subpopulation in this gate. Our results are expressed as the proportion of positive cells for the three markers in relation to the total number of gated cells.

### Clinical data

For each patient, we recorded the following data: demographics (age and sex); presence of traditional vascular risk factors including high blood pressure, diabetes mellitus, hypercholesterolemia, CAD, smoking habit, alcohol abuse, peripheral artery disease, atrial fibrillation, previous transient ischemic attack, previous cerebral infarct; treatment with any statin dose before the onset of stroke, during admission, and at 3 months. Also, we recorded whether this treatment was withdrawn. The routine practice in our stroke unit is to administer statins as soon as possible to every patients with ischemic stroke, and this treatment is usually indefinite; stroke etiological subtype, according to the SSS-TOAST classification (Ay et al. 2005); severity of the neurological deficit at admission (NIHSS score); mortality and functional outcome at 3 months. A favorable outcome was defined as a score 0–2 on the Rankin scale score.

### Statistical analyses

As we found many patients with a complete absence of EPC, we compared patients with (EPC+) and without EPC (EPC−). We combined patients with 1 or more EPC in the same EPC+ group. Using this dichotomized variable, we compared categorical variables with contingency tables and the Chi square test, and compared means and standard deviation of quantitative variables with the Student's *t*-test. NIHSS scores were compared with the Mann–Whitney *U* test. The time course of EPC counts was assessed with the analysis of variance (ANOVA) for repeated measures and the Greenhouse-Geisser test and confirmed with the nonparametric Friedman test. For most analyses, EPC counts were analyzed also as a continuous variable with nonparametric tests, as they did not follow a normal distribution (Mann–Whitney *U* test and Spearman's correlation were used).

To study the association of variables with EPC counts, a stepwise forward logistic regression analysis was performed by selecting variables with a *P*-value ≤ 0.1 in the bivariate analyses and by considering the EPC count as the dependent variable. All the analyses for the samples obtained at the acute (within 48 h), subacute (7 days), and chronic (3 months) stages were repeated. A similar approach was used to assess the prognostic value of EPC counts, with favorable outcome (Rankin 0–2) as the dependent variable.

## Results

From a total of 165 patients evaluated at baseline, 19 were excluded due to a diagnosis other than stroke, resulting in a final sample of 146 patients. The number of patients studied at day 7 was 121; no sample was available from the remaining due to death (*n* = 6), early discharge (*n* = 4), withdrawal of consent (*n* = 1), and defective blood sampling (*n* = 14). At the 3-month follow-up, we obtained a blood sample from 92 patients and we failed to collect a blood sample from 54 patients due to death (*n* = 9), withdrawal of consent (*n* = 1), information on functional outcome obtained by telephone (*n* = 21), defective blood sampling (*n* = 20), and unknown (*n* = 3). The demographic and clinical data are summarized in Table 1.

**Table 1:** Demographic and clinical characteristics of our patients

Variable	Baseline (*n* = 146)	Day 7 (*n* = 121)	3 months (*n* = 92)
Age (mean, SD)	70.8 (12.2)	70.1 (12.6)	69.1 (11.8)
Sex (% men)	63	65.3	68.5
Hypertension (%)	74.7	76	73.9
Diabetes mellitus (%)	31.5	28.1	20.7
Hypercholesterolemia (%)	39	35.5	38
Coronary artery disease (%)	20.5	19.8	18.5
Smoking (%)	30.8	33.1	33.7
Alcohol abuse (%)	6.2	5.8	5.4
Peripheral artery disease (%)	11.6	10.7	7.6
Atrial fibrillation (%)	24	23.1	22.8
Previous transient ischemic attack (%)	11	11.6	9.8
Previous cerebral infarction (%)	12.3	11.6	15.2
Statins (%)			
Pretreatment with statins	30.8	30.6	34.8
Statins during admission	–	94.2	94.5
Statins at 3 months^1^	–	–	95.3
Etiology (%)			
Large-artery atherothrombosis	14.4	14.9	13
Cardioembolism	38.4	40.5	43.5
Small-vessel disease	13.7	14	12
Other causes	3.4	4.1	4.3
Undetermined	30.1	26.4	27.2
NIHSS score at admission (median, interquartile range)	5.5 (10.5)	–	–

1 Information available in 85 of the 92 patients.

Overall, EPC levels were seen rarely in the peripheral blood (baseline: 0.002836 ± 0.0074482%; day 7: 0.007421 ± 0.137567%; 3 months: 0.004174 ± 0.1897642%); in fact, they were undetectable in about three quarters of the patients in the baseline (74.7%) and 3 months (77.2%) samples, and in about half of the patients in the 7-day sample (52.9%). Notably, the time-course analysis showed that circulating EPC count was significantly higher on day 7 than at baseline or day 90 (Greenhouse-Geisser test *P* = 0.045, Friedman test *P* < 0.001).

The association of variables with the EPC+ and EPC− groups is shown in Table 2. Most patients received statins during admission and were still receiving statins at 3 months. Withdrawal of statins occurred in only two patients, due to liver toxicity. Hypercholesterolemia (*P* = 0.034) and statin pretreatment (*P* = 0.025) were significantly more prevalent in the EPC+ group. Stroke of undetermined etiology was more frequent in the EPC+ group, and the large-artery atherothrombosis and cardioembolic subtypes were less frequent (global *P* = 0.017). As shown in Table 3, pretreatment with statins and stroke etiology were independent predictors of EPC+ at baseline. The same results were found using nonparametric tests for comparison of EPC counts (data not shown). No variables were associated with the EPC counts at day 7 and 3 months.

**Table 2:** Summary of the association between the EPC count and the variables listed in methods

	Baseline (*n* = 146)			7 days (*n* = 121)			3 months (*n* = 92)		
			
Variable^1^	EPC−	EPC+	*P*	EPC−	EPC+	*P*	EPC−	EPC+	*P*
Age (years)	70.5 (12.3)	71.7 (11.9)	0.60	69.4 (12.6)	70.9 (12.7)	0.52	69.9 (11.3)	66.3 (13.2)	0.23
Sex (% men)	63.3	62.2	0.99	64.1	66.7	0.84	66.2	76.2	0.43
Hypertension (%)	76.1	70.3	0.51	81.3	70.2	0.20	76.1	66.7	0.40
Diabetes mellitus (%)	30.3	35.1	0.68	28.1	28.1	0.99	19.7	23.8	0.76
Hypercholesterolemia (%)	33.9	54.1	0.034	35.9	35.1	0.99	38	38.1	0.99
Coronary artery disease (%)	20.2	21.6	0.81	17.2	22.8	0.49	19.7	14.3	0.75
Smoking (%)	28.4	37.8	0.46	35.9	29.8	0.42	33.8	33.3	0.96
Alcohol abuse (%)	8.3	0	0.11	4.7	7	0.70	4.2	9.5	0.32
Peripheral artery disease (%)	11.9	10.8	0.99	14.1	7	0.25	9.9	0	0.34
Atrial fibrillation (%)	22	29.7	0.37	20.3	26.3	0.51	23.9	19	0.77
Previous transient ischemic attack (%)	10.2	13.5	0.55	10.9	12.3	0.99	11.3	4.8	0.67
Previous cerebral infarction (%)	11.1	16.2	0.40	10.9	12.3	0.99	18.3	4.8	0.17
Statins (%)									
Prior to stroke	25.7	45.9	0.025	28.1	33.3	0.55	35.2	33.3	0.99
During admission	–	–		96.8	91.2	0.25	97.1	85.7	0.079
Statins at 3 months	–	–		–	–		95.5	94.7	0.99
Etiology (%)									
Large-artery atherothrombosis	15.6	10.8		20.3	8.8		12.7	14.3	
Cardioembolism	43.1	24.3	0.017	32.8	49.1	0.20	43.7	43.2	0.99
Small-vessel disease	12.8	16.2		17.2	10.5		12.7	9.5	
Other causes	0.9	10.8		3.1	5.3		4.2	4.8	
Undetermined	27.5	37.8		26.6	26.3		26.8	28.6	
Baseline NIHSS score (median, interquartile range)	6 (11)	4 (10.5)	0.97	6 (9.75)	6 (11)	0.78	6 (10)	9 (12)	0.25
Rankin score 0–2 (%)	61.5	73	0.23	62.5	66.7	0.70	73.2	57.1	0.18

1 Categorical variables are presented as percentage.

**Table 3:** Logistic regression analysis of the influence of stroke etiology on EPC counts

Variable	OR	CI 95%	*P-*value
Pretreatment with statins	3.11	1.34–7.19	0.008
Stroke etiology (undetermined etiology = 1)			0.032
Undetermined	–		
Large-artery atherothrombosis	0.507	0.139–1.849	0.304
Cardioembolism	0.342	0.126–0.929	0.035
Small-vessel disease	0.882	0.270–2.887	0.836
Other causes	10.266	1.012–104.11	0.002

Median baseline NIHSS scores were equivalent between EPC+ and EPC− groups at the three time points (Table 2). Moreover, no correlation was found between the baseline NIHSS scores and the EPC counts at baseline, day 7, and 3 months. At the 3-month follow-up, 94 patients (64.4%) had a favorable outcome, 43 (29.4%) scored 3–5 in the Rankin scale, and 9 patients (6.2%) had died. As shown in Table 2, the proportion of patients with a favorable outcome was the same in patients with or without EPC, either at baseline, day 7 and 3 months. Also, nonparametric correlations between EPC counts and Rankin scores were not statistically significant. The evaluation of mortality yielded nonsignificant differences also.

However, when considering the stroke etiology, EPC counts at baseline showed important prognostic results in some subgroups. Combining the two groups of arterial origin (large-artery atherothrombosis and small-vessel patients, *n* = 41) the frequency of favorable outcome in patients with EPC+ counts at baseline was 10/10 (100%), while it was 19/31 (61.3%) in patients from the EPC− group (*P* = 0.021). This association was not found for samples obtained at day 7 or 3 months. A logistic regression analysis with favorable outcome as the dependent variable in which age and NIHSS score were used as prognostic outcome variables showed that only age (OR 0.90, confidence interval [CI] 95% 0.83–0.98, *P* = 0.015) and EPC+ group (*P* = 0.021, undefined OR due to the lack of cases in a cell) independently predicted outcome.

## Discussion

We evaluated the counts of circulating EPC at different stages in patients with ischemic stroke. We found that the levels of circulating EPC peaked at day 7, but were absent in nearly half of the patients; prior treatment with statins and stroke etiology were significantly associated with the counts of EPC at the acute stage. Finally, although EPC counts were neither related to the severity of the neurological deficit nor to the outcome, a favorable prognosis at 3 months was associated with the EPC+ counts in patients with large-artery atherothrombosis or with small-vessel disease.

EPC are extremely rare in the peripheral blood of adults. They account for 0.0001–0.01% of mononuclear cell (Ingram et al. 2005) and the true normal values are equivocal. We found very low EPC counts in our patients, and at day 7 EPC were detected by flow cytometry in only about 50% of patients. To feel confident that our results were reliable, we acquired a minimum of 300,000 events for each sample. Other authors (Cesari et al. 2009; Bogoslovsky et al. 2011) who used flow cytometry also found very low counts in patients with acute ischemic stroke. These very low or absent counts may be explained by the lack of production of EPC in the bone marrow, an increased utilization of these cells at sites that require vascular repair, or a reduced half-life of circulating EPC.

After the ischemic injury, the release of cytokines and trophic factors may induce an increased production and mobilization of EPC (Rouhl et al. 2008). This occurs in patients with acute coronary syndrome (Shintani et al. 2001) and acute ischemic stroke (Zhou et al. 2009) with a peak of EPC counts and Vascular endothelial growth factor (VEGF) levels (Sobrino et al. 2012a) at 7 days after the ischemic event. In our study, we confirmed the increase at day 7 in comparison with the baseline and 3-month measurements. However, one study (Ghani et al. 2005) reported stable EPC counts while another study (Dunac et al. 2007) reported an intermittent release of EPC after ischemic stroke.

Our finding of very low or absent EPC counts agree with three studies (Ghani et al. 2005; Chu et al. 2008; Zhou et al. 2009) that reported lower EPC counts in patients with acute ischemic stroke compared to healthy controls. However, the data are inconsistent as other authors found higher EPC counts in patients than in controls (Dunac et al. 2007; Yip et al. 2008, 2011; Navarro-Sobrino et al. 2010). Different patient characteristics (such as age or distribution of risk factors), time from stroke onset to blood collection, EPC definitions, EPC measurements, and statistical methods may account for these discrepancies. In healthy subjects and in patients with CAD, increasing age and traditional vascular risk factors adversely affect EPC levels and function (Hill et al. 2003; Werner et al. 2005; Fadini et al. 2007; Rouhl et al. 2008), although other studies reported conflicting results (Eizawa et al. 2004; Kunz et al. 2006; Hristov et al. 2007; Xiao et al. 2007). Conversely, statins, estrogen, erythropoietin, angiotensin-converting enzyme inhibitors and physical exercise tend to increase EPC counts (Werner et al. 2005; Fadini et al. 2007). Clearly, as EPC may play an important role in the pathophysiology of ischemic stroke, it is worthwhile to investigate the variables that influence the levels of these cells. It is possible that these variables have prognostic and therapeutic consequences. In our study we did not observe an influence of aging or vascular risk factors on EPC counts. Only patients who received prior treatment with statins and specific etiologies were significantly associated with EPC counts. A direct comparison of our study with previous studies is not possible for several reasons: statin pretreatment was not included as a variable (Ghani et al. 2005; Chu et al. 2008; Zhou et al. 2009); etiology subtype was not analyzed (Ghani et al. 2005; Yip et al. 2008; Zhou et al. 2009); the time from stroke onset to time of blood sampling were not restricted to the acute stage (Chu et al. 2008) or was not provided (Ghani et al. 2005); and the number of recruited patients was relatively small (Ghani et al. 2005; Chu et al. 2008; Cesari et al. 2009). Additionally, some studies used flow cytometry (Yip et al. 2008; Cesari et al. 2009; Zhou et al. 2009) while others relied on counting colony-forming units (Ghani et al. 2005; Chu et al. 2008). Also, the definition of EPC was variable among the studies (Ghani et al. 2005; Chu et al. 2008; Yip et al. 2008; Zhou et al. 2009). To our knowledge, our study is the largest to date and the only one that analyzed serial samples at the acute, subacute, and chronic stage of stroke.

Statins have several effects that are beneficial for patients with acute ischemic stroke, and are independent of the lipid-lowering properties (Marti-Fabregas et al. 2004). These effects may be mediated by the increase in the mobilization and the improvement of the functional activity of the EPC population, that has been demonstrated in vitro and in patients with stable ischemic heart disease (Vasa et al. 2001; Urbich and Dimmeler 2004). Thus, this influence of statins is likely a novel pleiotropic effect. The administration of statins to patients with stable CAD increases the number of EPC (Vasa et al. 2001), but these results were not replicated in patients with chronic stroke (Mohammad et al. 2010). Recently, a study in patients with acute ischemic stroke reported that statin treatment for 4 days may increase circulating EPC levels (Sobrino et al. 2012b). In our study, the effect of statins on EPC number was limited to the acute stage, with a trend to fewer EPC in the chronic stage, a paradoxical effect also reported in patients with CAD, and attributed to exhausted mobilization, pro-apoptotic effects, enhanced recruitment, or improved neo-endothelial incorporation of EPC.

The production of EPC after ischemic stroke probably augments the repair of the damaged endothelium, may help to reestablish the blood–brain barrier, to decrease the risk of recurrence and to promote the formation of collaterals and neovascularization. It seems likely that these functions entail a favorable prognostic value in acute stroke (Dunac et al. 2007; Sobrino et al. 2007; Yip et al. 2011). In our study, we did not find an association between EPC counts and outcome in the whole sample of patients, but significantly, after evaluating the prognosis in etiological subgroups, we found that those with artery-related stroke etiology in whom EPC were present had a better outcome than patients with no EPC. This finding has not been reported by previous studies: one study excluded lacunar subtype (Sobrino et al. 2007), another study focused on “atherothrombotic” patients (Dunac et al. 2007), and in a third study etiology was not evaluated (Yip et al. 2011). Endothelium damage leads to bone marrow exhaustion or to a greater EPC repair activity, and therefore to lower peripheral blood levels. In support of our findings, damaged endothelium is typically found in atherosclerosis (Ross 1999) and plays also an essential role in lacunar strokes (Hassan et al. 2003). Patients with cerebral autosomal dominant arteriopathy with subcortical infarcts and leukoencephalopathy (CADASIL; Pescini et al. 2010) and with leukoaraiosis (Jickling et al. 2009), that are considered manifestations of small-vessel disease with dysfunctional endothelium, have lower levels of EPC also.

As in our study and others (Zhou et al. 2009; Yip et al. 2011; Sobrino et al. 2012a) the severity of stroke was not associated with EPC counts, we believe that higher EPC counts do not lead to milder strokes. The fact that only EPC measured within the first 48 h were associated with prognosis suggests that the positive effect of EPC is exerted very soon after the onset of stroke.

Our study has some limitations. Undoubtedly, our analyses of association between EPC counts and different variables and between EPC counts and prognosis would have been strengthened by a larger sample. We did not include a control population. Also, we did not know the doses, length of treatment, and compliance of statin treatment prior to admission. We measured the amount of EPC, but not their function; however, amount and functional integrity are coregulated by the same molecular pathways (Fadini et al. 2007), so that a decrease in EPC numbers is usually associated with a decrease in EPC function, and vice versa. The lack of a consensus on the optimal definition of EPC and on the best method to measure this cell population (Rouhl et al. 2008) may be responsible for the discrepancies among various studies. We used flow cytometry analysis in combination with markers CD34, CD133, and KDR because flow cytometry and the use of markers are highly recommended and widely used (Urbich and Dimmeler 2004) and undoubtedly identify and count EPC unequivocally.

In summary, our findings indicate that EPC are rarely seen in the peripheral blood of patients with acute ischemic stroke and we confirmed an increase of EPC levels in the subacute stage. Significantly, patients who were receiving statins at the time of stroke had higher EPC levels. The presence of EPC may improve the outcome of certain stroke subtypes, that is, large-artery atherothrombosis and small-vessel disease. We consider that the precise mechanisms by which EPC are associated with outcome deserve further studies. Further studies should explore whether EPC may have a therapeutic role in ischemic stroke.
